# A genome-wide scan study identifies a single nucleotide substitution in *MC1R* gene associated with white coat colour in fallow deer (*Dama dama*)

**DOI:** 10.1186/s12863-020-00950-3

**Published:** 2020-11-19

**Authors:** Gerald Reiner, Tim Weber, Florian Nietfeld, Dominik Fischer, Christine Wurmser, Ruedi Fries, Hermann Willems

**Affiliations:** 1 Department for Veterinary Clinical Science, Justus-Liebig-University, Frankfurter Strasse 112, D-35392 Giessen, Germany; 2 Arbeitskreis Wildbiologie e.V., Justus-Liebig-University, Giessen, Germany; 3 Department of Animal Breeding, Technical University of Munich, Liesel-Beckmann-Strasse 1, D-85354 Freising-Weihenstephan, Germany

**Keywords:** Fallow deer, White coat colour, *MC1R*, Next generation sequencing

## Abstract

**Background:**

The coat colour of fallow deer is highly variable and even white animals can regularly be observed in game farming and in the wild. Affected animals do not show complete albinism but rather some residual pigmentation resembling a very pale beige dilution of coat colour. The eyes and claws of the animals are pigmented. To facilitate the conservation and management of such animals, it would be helpful to know the responsible gene and causative variant. We collected 102 samples from 22 white animals and from 80 animals with wildtype coat colour. The samples came from 12 different wild flocks or game conservations located in different regions of Germany, at the border to Luxembourg and in Poland. The genomes of one white hind and her brown calf were sequenced.

**Results:**

Based on a list of colour genes of the International Federation of Pigment Cell Societies (http://www.ifpcs.org/albinism/), a variant in the *MC1R* gene (NM_174108.2:c.143 T > C) resulting in an amino acid exchange from leucine to proline at position 48 of the MC1R receptor protein (NP_776533.1:p.L48P) was identified as a likely cause of coat colour dilution. A gene test revealed that all animals of the white phenotype were of genotype CC whereas all pigmented animals were of genotype TT or TC. The study showed that 14% of the pigmented (brown or dark pigmented) animals carried the white allele.

**Conclusions:**

A genome-wide scan study led to a molecular test to determine the coat colour of fallow deer. Identification of the *MC1R* gene provides a deeper insight into the mechanism of dilution. The gene marker is now available for the conservation of white fallow deer in wild and farmed animals.

**Supplementary Information:**

The online version contains supplementary material available at 10.1186/s12863-020-00950-3.

## Background

White coat colour or dilution are commonly found within fallow deer in game farming and in the wild. It is important for the management of the white animals to identify the responsible gene variant and develop a gene marker. This is the only way to make informed statements about the distribution of the white gene allele in a population. However, up to now, no scientific articles have been available on the colouring of fallow deer and nothing was known about the genes that are responsible for the white coat colour in this species.

A list of 256 genes involved in the white colour or dilution is available from the International Federation of Pigment Cell Societies (http://www.ifpcs.org/albinism/). The most important proteins are formed in melanocytes where they are involved in pigmentation on five independent levels: melanocyte development and migration, melanosome biogenesis, melanosome transport, biosynthesis of melanin and control of melanogenesis.

Major proteins involved in melanocyte development and migration are the tyrosinase protein kinase KIT (KIT), the KIT ligand (KITLG), endothelin 3 (EDN3), endothelin receptor type b (EDNRB). Melanocytes contain melanosomes, subcellular lysosome-like organelles in which melanin pigments are synthesized and stored before distribution to the surrounding keratinocytes. Biogenesis of melanosomes is controlled by premelanosome protein (PMEL), silver (SILV), pink-eyed dilution protein (P) and adaptor related protein complex 3 (AP3). Melanophilin (MLPH), myosin Va (MYO5A) and RAS-related protein b27a (RAB27A) are involved in melanosome transport.

Tyrosinase (TYR), tyrosinase-related protein 1 (TYRP1) and dopachrome tautomerase (DCT) are involved in the biosynthesis of the different kinds of melanin [1]. TYR catalyses the rate-limiting reaction in melanin synthesis, converting tyrosine to dopaquinone and oxidizing 5,6-dihydroxyindole (DHI) to indole-5,6-quinone [2]. TYRP1 and DCT function further downstream in the melanin biosynthetic pathway [2, 3]. Melanin synthesis is regulated by α-melanocyte stimulating hormone derived from pro-opiomelanocortin 1 (POMC1), melanocortin 1 receptor (MC1R), agouti signalling protein (ASIP), microphthalmia-associated transcription factor (MITF) and by additional proteins such as the premelanosome protein 17 (PMEL17) [4], the pink-eyed dilution protein [5], and the melanoma antigen recognized by T-cells protein (MART-1) [6]. The MC1R and its ligand, the α-melanocyte stimulating hormone (α-MSH) are involved in modifications of coat colour [7]. Further factors involved in transcriptomic regulation are the MITF, a basic-helix-loop-helix (bHLH) transcription factor [8].

In other ruminants, for example cattle, at least eight different genes have been associated with white colouring: *ASIP* [9], *TYR* [10], *KIT* [11], *KITLG* [12], *MC1R* [13, 14], *PMEL* [14], mast cell growth factor (*MGF*) [15] and *MITF* [16].

In addition to colour inheritance in cattle [17], information is also available on sheep [18], goats [19] and buffalos [20]. Recently, we characterized a single nucleotide substitution in the *TYR* gene associated with white coat colour in a red deer (*Cervus elaphus*) population [21]. While variants in tyrosinase are commonly associated with oculocutaneous albinism type 1, an amino acid exchange at position 291 in TYR was found to be associated with coat colour dilution in this population.

Nothing is known about colour inheritance in *Dama dama*. Although so far only a few genes seem to be associated with the whitening of cattle, there is still a wide range of candidate genes to be considered in the search for the genetic cause of the whitening of fallow deer.

The aim of the present study was therefore to identify the causative variant for coat colour dilution in the fallow deer and to develop a genetic marker to facilitate the conservation of white animals.

## Results

**Fig. 1:**
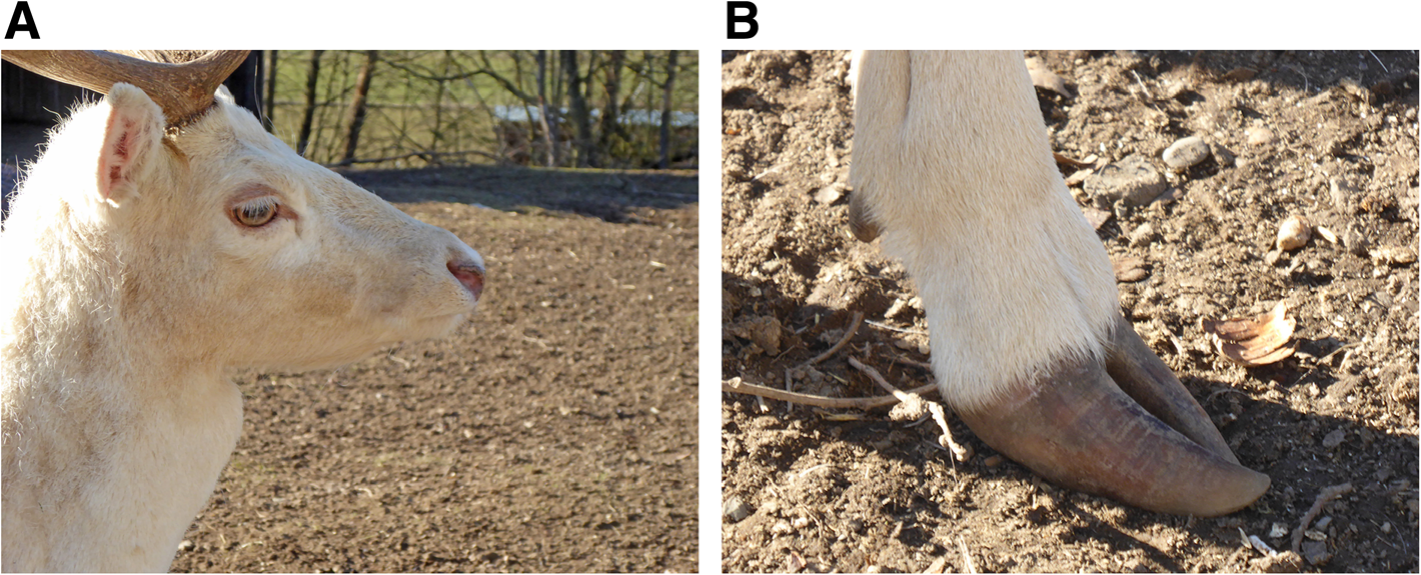
Phenotype of the coat, eye and muzzle (**a**) and the claws (**b**) of a typical white individual

Whole genome sequencing of a white hind (Fig. 1) and her brown calf was performed to reveal the causative variant of colour dilution in fallow deer.

Sequencing of the hind and her calf resulted in a coverage of 9.48 and 9.68 fold, respectively. A total of 26.18 and 26.71 gigabases were sequenced. 85.58 and 85.69% of these sequences could be mapped to the bovine genome, respectively. Around 11 million SNPs were identified.

After variant calling and annotation, 12,751 SNPs were extracted as a subset of SNPs based on a list of colour genes detected in mice, human and zebrafish (International Federation of Pigment Cell Societies; http://www.ifpcs.org/albinism/). Three thousand nine hundred fifty-three of them were non-synonymous (ns) SNPs that covered 149 genes. They were located in *ASIP* (2 ns + 5 synonymous [s] SNPs), *DCT* (21 ns + 33 s SNPs), *EDNRB* (6 ns + 15 s SNPs), *KIT* (18 ns + 85 s SNPs), *MC1R* (11 ns + 49 s SNPs), *TYR* (20 ns + 45 s SNPs) and *TYRP1* (25 ns + 46 s SNPs). Synonymous SNPs were excluded from further processing. Following the hypothesis of a recessive inheritance of the white colour, we expected the genotype of the white hind to be homozygous for the white allele and the brown calf to be heterozygous. All genes and SNPs that did not correspond to this assumption were filtered out.

After filtering, sixteen genes with nineteen non-synonymous SNPs were left and confirmed by Sanger sequencing (Table 1). For each of these SNPs a PCR system was established to test the association of the gene variant with the phenotypes of one additional white and one additional brown individual of the population. Four SNPs showed the expected genotype-phenotype association and were tested on further white (*n* = 3) and brown (*n* = 3) individuals. Only the genotypes at one SNP in the *MC1R* gene were associated to the phenotype in these individuals. Results of the phenotype-genotype association are presented in Supplementary Table 1. Subsequently all animals (*n* = 102) were tested for the SNP at the *MC1R* gene revealing a 100% match between genotype and phenotype (Fisher’s exact test: *P* = 8.4^− 23^) (Table 2).

**Table 1:** List of candidate genes after extracting non-synonymous colour genes and testing with Sanger sequencing

Chr	Chr_pos	Ref	Alt	Gene	Acc.No.	SNP	Codon	aa subst.
13	51,640,322	C	T	*ATRN*	NM_173995.3	c.1573C > T	Gat/Aat	p.D525NN
23	41,483,931	G	C	*DTNBP1*	NM_001045947.1	c.871G > C	Gcc/Ccc	p.A291P
26	22,870,530	A	G	*ELOVL3*	NM_001192306.1	c.73A > G	Aac/Gac	p.N25D
12	23,298,094	C	T	*FREM2*	XM_002691799.5	c.9260C > T	aGg/aAg	p.R3087K
12	23,336,198	G	T	*FREM2*	XM_002691799.5	c.6042G > T	gaC/gaA	p.D2014E
17	71,465,091	T	C	*GGT1*	NM_001206214.1	c.100 T > C	Agc/Ggc	p.S34G
28	4,036,781	G	A	*GNPAT*	NM_001103286.1	c.1462G > A	Gtt/Att	p.V488I
X	37,080,066	C	T	*L1CAM*	NM_001192435.1	c.2722C > T	Ggg/Agg	p.G908R
28	8,475,854	A	C	*LYST*	NM_174020.2	c.5337A > C	ttA/ttC	p.L1779F
18	14,705,518	T	C	*MC1R*	NM_174108.2	c.143 T > C	cTg/cCg	p.L48P
11	103,981,017	T	G	*NOTCH1*	XM_024999642.1	c.43 T > G	Aca/Cca	p.T15P
3	13,984,639	T	C	*NTRK1*	XM_024989930.1	c.55 T > C	Agg/Ggg	p.R19G
5	57,353,185	T	C	*PMEL*	NM_001080215.2	c.1873 T > C	Tgg/Cgg	p.W625R
14	429,568	A	G	*RECQL4*	NM_001098037.2	c.370A > G	Acc/Gcc	p.T124A
14	429,631	T	C	*RECQL4*	NM_001098037.2	c.433 T > C	Tca/Cca	p.S145P
4	40,075,565	G	A	*SEMA3C*	NM_001101082.1	c.718G > A	Gtg/Atg	p.V240M
3	14,622,613	C	G	*SEMA4A*	NM_001075440.1	c.320C > G	aGt/aCt	p.S107T
X	133,162,026	G	T	*SHROOM2*	XM_002700461.5	c.3176G > T	gCc/gAc	p.A1059D
X	133,162,029	G	A	*SHROOM2*	XM_002700461.5	c.3173G > A	tCc/tTc	p.S1058F

*Chr Bos taurus* reference chromosome; *Chr_pos* position on the bovine chromosome (in bp); *Ref* reference nucleotide; *Alt* alternative nucleotide; *Gene* gene name; *Acc.No*. accession number of the NCBI reference sequence; *SNP* polymorphism in the NCBI reference sequence; *Codon* codon with SNP in capital letters; *aa subst*. amino acid substitution (according to the protein_id given in the NCBI reference sequence)

**Table 2:** Association between phenotype and *MC1R* genotype in 102 examined individuals from 12 locations

	MC1R-SNP (c.143 T > C) genotype		
Coat colour	TT	TC	CC
WT/dark pigmented	69	11	0
White	0	0	22

Indicated are the number of animals with the respective coat colour and genotype; *WT* wildtype; Fisher’s exact test: *P* = 8.4^−23^)

Eleven out of 80 wildtype or dark pigmented individuals carried the TC genotype (14%). Any other wildtype or dark pigmented individual were homozygous for the T-allele. Any of the white individuals was homozygous for the C-allele. The *MC1R* SNP (c.143 T > C) is predicted to result in an amino acid substitution from leucine to proline (p.L48P).

## Discussion

Although a complete genome sequence of *Dama dama* is not available, the high degree of sequence conservation, even of microsatellites, between cervides and other ungulates, especially cattle [22, 23] led us to attempt to map genomic fallow deer sequences to the bovine reference genome. Indeed, fallow deer sequences homologous to 86% of the bovine genome were mapped, revealing 11 million SNPs. We were confident that the coding sequence regions in particular would show a good match between fallow deer and bovine genome. In a study on white coat colour in *Cervus elaphus* [19] 82% of *Cervus* sequences were mapped based on the well-established bovine genome (UMD 3.1, Ensembl release 94, NCBI assembly accession GCA_000003055.3), as compared to 92% when mapped to the *Cervus elaphus* genome CeEla1.0 [24]. Since the *TYR* gene that is responsible for the white phenotype in the studied red deer population was not annotated in CerELa1.0, there was no chance that it would be detected based on the *Cervus* reference sequence. This is not unexpected, since 21,880 genes are annotated for the bovine genome in contrast to 19,368 for the genome of *Cervus elaphus*. Therefore, we decided to use the better annotated bovine genome UMD 3.1. In fact, more than 12,000 SNPs were extracted after variant calling as a subset based on a list of colour genes (International Federation of Pigment Cell Societies; http://www.ifpcs.org/albinism/). Nineteen SNPs in sixteen candidate genes corresponded exactly to the requirements of a homozygous white hind and its heterozygous brown calf. However, only one SNP, located in the *MC1R* gene showed perfect genotype-phenotype association in the entire cohort with 22 white and 80 brown individuals, collected from 12 locations with unrelated fallow deer populations, mostly in Germany. However, we have to admit that the method used would probably not have found all types of variants, especially those in gene regions with lower agreement between *Dama dama* and *Bos taurus*, e.g. in non-coding regions. Furthermore, indels and large structural variants would not have been detected by our approach.

The *MC1R* gene is involved in a huge network of colouring genes (for overview see [25]), and thus associated with a broad spectrum of colour variation in human [26], mouse [27] and several other mammalian and bird species, such as horses [28], foxes [29], dogs [30], rabbits [31], chicken [32], alpacas [33], buffalos [20], sheep [34, 35], goats [36], and cattle [37, 38]. Variants were described together with the prevention of the white winter coat in foxes [39] and increased pigmentation in reindeer [40] and other species (overview by [41]). However, besides oculocutaneous albinism type 2 in humans [26], associations of *MC1R* variants with white coat colour are rare. They were found in black bears [42], white leghorn chicken [43], martens [44], mice [45], Huskies [46] and the Arabian camel [47]. MC1R has never been associated with colour variation or dilution in cervids.

MC1R is a seven-pass transmembrane G protein [26] coupled receptor that is especially located on the surface of melanocytes. MC1R is activated by the α-melanocyte stimulating hormone (α-MSH) and competitively inhibited by the agouti signalling protein (ASIP). Activation stimulates an adenylate cyclase and increases the amount of cAMP, activating the transcription of enzymes involved in eumelanin production [26], e.g. TYRP1 and TYR, the key enzymes in melanin biosynthesis [2, 48]. The loss of function of MC1R because of sequence variation affects the ability to generate cAMP and leads to minimal production of eumelanin in melanocytes. Variation within the transmembrane helices can result in loss of function. The variation which is responsible for white coat colour in fallow deer was detected at nucleotide 143 (c.143 T > C) that leads to an amino acid exchange from leucine to proline (p.L48P) in the present study. This substitution is located within the helix structure of the first transmembrane motif, where several non-synonymous variants have been described in humans (V38M, S41F, V51A) [49], I40T [50], and V60L [51] (for overview see [26]). These variants resulted in reduced cell surface expression of MC1R as a consequence of retention in the endoplasmic reticulum (V38M, S41F, V51A) and/or with a decreased coupling to adenylate cyclase (V60L). Although the variant of the fallow deer has never been described before, it is closely related to the above-described human variants. While leucine, the wild-type amino acid is a typical component of α-helices, introduction of a proline residue into similar membrane-bound proteins was shown to alter the gross secondary structure from α-helix to ß-sheet-like [52], which could be detrimental to the structure. Because of its very rigid structure, which bends the main chain of the protein in a characteristic way, proline is a well-known breaker of secondary structures [53, 54]. In contrast to proline, leucine is found with above-average frequency in helix structures and is very rarely replaced by other amino acids, an indication of the important structural function this amino acid occupies there [54]. Further studies are necessary to prove the functional significance of the p.L48P variant in white fallow deer, especially in populations from other parts of the world.

## Conclusion

The genomic sequencing of a white hind and her brown calf led to the identification of a non-synonymous variant with exchange of a leucine residue at position 48 of the melanocortin 1 receptor by proline as a likely cause of dilution of the coat colour. This variant was detected using a list of colour genes of the International Federation of Pigment Cell Societies (http://www.ifpcs.org/albinism/). Genetic testing confirmed the expected genotypes in all 22 white and 80 brown animals from 12 different locations examined. The study showed that 14% of the brown animals carry the white allele. This genetic test provides a simple and reliable way of conservation and management for the white animals.

## Methods

### Fallow deer

Samples of fallow deer were collected from 12 locations. Four locations were hunting grounds and eight locations were game parks or game farms (Table 3). The different locations contributed between 1 and 29 animals, 21.6% of which had a white coat colour. Samples were taken from existing antlers and frozen tissue samples provided either by official game parks or by those authorised to practise hunting. No animals were killed specifically for the study. No live animals were sampled and no dropping antlers were sought or collected for the study.

**Table 3:** Origin of fallow deer samples

Location	n	Origin	Coat colour (n)	
			WT/dark pigmented	white
Edersee, Germany, 52.957399, 12.851258	13	G	10	3
Eulbach, Germany, 49.682958, 9.067647	22	G	19	2
Griebelschied, Germany, 49.801959, 7.393967	2	G	0	2
Hanstedt, Germany, 53.238308, 10.046076	6	G	1	5
Murowana, Poland, 52.406374, 16.9251681	1	H	1	0
Weishauswald, Germany, 49.771825, 6.629594	6	G	5	1
Müritz, Germany, 53.473235, 12.798000	3	H	2	1
Neuruppin, Germany, 52.957399, 12.851258	13	H	13	0
Pleizenhausen, Germany, 50.015735, 7.564419	1	G	0	1
Sababurg, Germany, 51.545171, 9.532297	29	G	24	5
Weilburg, Germany, 50.488850, 8.332017	2	G	2	0
Wolfshagen, Germany, 54.191712, 12.821344	4	H	3	1
Total number of animals	102		80	22

*Location* region, country, latitude; altitude; *n* number of samples; *Origin* game park (G) or hunting ground (H); *WT* wildtype brown

### The phenotype

The white fallow deer were not albinos, but the coat colour was diluted. There were no noticeable differences in the degree of dilution. The eyes and claws were normally pigmented or slightly lightened. Apart from the coat and eye colour, the white animals did not differ from the brown animals in size, weight or habitus.

### Sample collection

Samples from pigmented (normal brown and dark pigmented, *n* = 80) and white (*n* = 22) fallow deer were collected over the 2017/2018 seasons. Two female animals (one white adult hind with its brown calf) were available for Next Generation Sequencing. All samples were accompanied with information about age, weight, colour, and hunting ground.

Samples from antlers were taken as drill core samples from the base and stored dry at ambient temperature. Tissue samples from meat were frozen at − 20 °C until use.

### DNA extraction

Genomic DNA was extracted from tissue samples and antler drill cores with the Instant Virus RNA Kit (Analytik Jena, Germany). Antler drill cores (0.1 to 0.3 g) were treated in a bead mill (MM200, Retsch, Germany) at a frequency of 25 Hz for 2 min. Tissue samples were suspended in 450 μl of lysis buffer and subsequently treated in the same way as the antler drill cores. All following steps were performed according to the manufacturer’s instructions. The extracted DNA was eluted with 60 μl of RNAse-free water.

DNA concentration was measured photometrically with the Nanodrop 2000 spectrophotometer (Thermofisher, USA) and the Qubit 2 system (Qubit dsDNA br assay kit and Qubit dsDNA hs assay kit, Thermofisher, USA).

### Next generation sequencing

DNA samples of one white female and it’s brown offspring were used for library preparation with the TruSeq DNA PCR-free sample preparation kit (Illumina, USA). The protocol was adjusted to receive fragments with a 350 bp insert size according to the manufacturer’s instructions. Quantification and quality control of the libraries was carried out through qPCR with the Kapa Library Quantification Kit (Kapa Biosystems, USA) and high-resolution electrophoresis with the Bioanalyzer 2100 (Agilent Genomics, USA). Paired-end sequencing with a read length of 2 × 126 bp was performed on a HiSeq2500 (Illumina, USA) with HiSeq v4 chemistry.

Raw data were demultiplexed and .*fastq* files were generated with bcl2fastq Conversion Software (Illumina, USA). The quality of the sequence reads was observed by *FastQC* (http://www.bioinformatics.babraham.ac.uk/projects/fastqc/).

All programs used in further processing of raw reads were embedded in python scripts to connect the different steps and programs.

In a first step raw sequences were converted from a base call file (bcl) to fastq files and mixed probes were demultiplexed through the program bcl2fastq Conversion Software from Illumina (http://emea.support.illumina.com/downloads/bcl2fastq_conversion_software_184.html?langsel=/de/). Because a *Cervus elaphus* reference genome was not available at the time of sequencing, resulting reads were aligned to the reference sequence of the bovine genome (UMD 3.1 [55]) using the *BWA-MEM* algorithm (https://arxiv.org/abs/1303.3997). After processing of data, single files were merged and converted from the *SAM* to the *BAM* format with *SAMtools*^56^. Duplicated reads were marked by the *PICARDtools* command *MarkDuplicates* (https://github.com/broadinstitute/picard/).

### Variant calling, annotation and identification of candidate variants

To identify single nucleotide polymorphisms (SNPs) in the annotated reads of the two sequenced fallow deer samples, we used the *mpileup* algorhithm implemented in *SAMtools* [56]. With the *filter* algorithm from *PICARDtools* (https://github.com/broadinstitute/picard/) called variants were filtered by excluding all SNPs within 3 base pairs of an INDEL and with lower *QUAL* score.

For the functional annotation of each called SNP we adapted the *VariantEffectPredictor (VEP)* from Ensemble [57].

Furthermore, we extracted a subset of SNPs based on a list of colour genes detected in mice, humans and zebrafish (International Federation of Pigment Cell Societies; http://www.ifpcs.org/albinism/). The resulting *VEP* annotated files containing only genomic regions coding for coat colour were checked on the basis of a recessive genetic inheritance model for non-synonymous impacts of the variants.

### Validation of candidate SNPs

SNPs detected by NGS were validated by Sanger sequencing (LGC Genomics, Germany). For this purpose, regions including the candidate SNPs were PCR amplified and sequenced. PCR primers were designed from the NGS data in combination with data from the *Bos taurus* reference genome.

## Supplementary Information

**Additional file 1:** **Supplementary Table S1.** Genotype-phenotype associations in white and brown fallow deer for all tested single nucleotide variants.

## Data Availability

Data and materials are available from the authors on reasonable request. Sequence data are available in the European Nucleotide Archive, Accession number PRJEB38970.

## Abbreviations

°C: Degrees centigrade
μl: Microliter
μM: Micromolar
A: Adenine
AP3: Adaptor related protein complex 3
ASIP: Agouti signalling peptide
bHLH: Basic-helix-loop-helix
bp: Basepairs
CerEla 1.0: Reference genome of *Cervus elaphus*
DCT: Dopachrome tautomerase
DHI: 5,6-dihydroxyindole
DNA: Desoxyribonucleic acid
EDNRB: Endothelin receptor type b
EDN3: Endothelin 3
g: Gram
G: Guanine
Hz: Hertz (1/s)
INDEL: Insertion-deletion mutation
KIT: Tyrosine protein kinase KIT
KITLG: KIT ligand
MART-1: Melanoma antigen recognized by T-cells
MC1R: Melanocortin 1 receptor
MGF: Mast cell growth factor
MITF: Microphthalmia-associated transcription factor
MLPH: Melanophilin
MYO5A: Myosin Va
n: Number
ng: Nanogram
ns: Non-synonymous
OCA1: Oculocutaneous albinism type 1
P: Pink-eyed dilution protein
PCR: Polymerase chain reaction
PMEL: Premelanome protein
PMEL17: Premelanosome protein 17
POMC1: Pro-opiomelanocortin 1
qPCR: Quantitative PCR
RAB27A: RAS-related protein b27a
s: Synonymous
SILV: Silver
TYR: Tyrosinase
TYRP1: Tyrosinase related protein 1
UMD 3.1: *Bos taurus* reference genome
α-MSH: α-melanocyte stimulating hormone

## References

[1] Kushimoto T, Valencia JC, Costin GE, Toyofuku K, Watanabe H, Yasumoto K (2003). The Seiji memorial lecture: the melanosome: an ideal modelt o study cellular differentiation. Pigment Cell Res.

[2] Ito S, Wakamatsu K, Ozeki H (2000). Chemical analysis of melanins and its application to the study of the regulation of melanogenesis. Pigment Cell Res.

[3] Lamoreux ML, Wakamatsu K, Ito S (2001). Interaction of major coat colour gene functions in mice as studied by chemical analysis of eumelanin and pheomelanin. Pigment Cell Res.

[4] Chakraborty AK, Platt JT, Kim KK, Kwon BS, Bennett DC, Pawalek JM (1996). Polymerization of 5,6-dihydroxyindole-2-carboxylic acid to melanin by the pmel 17/silver locus protein. Eur J Biochem.

[5] Brilliant MH (2001). The mouse p (pink-eyed dilution) and human P genes, oculocutaneous albinism type 2 (OCA2), and melanosomal pH. Pigment Cell Res.

[6] Aydin IT, Hummler E, Smit NPM, Beermann F (2011). Coat colour dilution in mice because of inactivation of the melanoma antigen MART-1. Pigment Cell Mel Res.

[7] Abdel-Malek Z, Suzuki I, Tada A, Im S, Akcali C (1999). The melanocortin-1 receptor and human pigmentation. Ann N Y Acad Sci.

[8] Tachybana M (2000). MITF: a stream flowing for pigment cells. Pigment Cell Res.

[9] Albrecht E, Komolka K, Kuzinski J, Maak S (2012). Agouti revisited: transcript quantification of the ASIP gene in bovine tissues related to protein expression and localization. PLoS One.

[10] Schmutz SM, Berryere TG, Ciobanu DC, Mileham AJ, Schmidtz BH, Fredholm M (2004). A form of albinism in cattle is caused by a tyrosinase framesift mutation. Mamm Genome.

[11] Fontanesi L, Tazzoli M, Russo V, Beever J (2010). Genetic heterogeneity at the bovine KITgene in cattle breeds carrying different putative alleles at the spottinglocus. Animal Gen..

[12] Charlier C, Denys B, Belanche JI, Coppieters W, Grobet L, Mni M (1996). Microsatellite mapping of the bovine roan locus: a major determinantof white heifer disease. Mamm Genome.

[13] Mohanti TR, Seo KS, Park KM, Choi TJ, Choe HS, Baik DH (2008). Molecular variation in pigmentation genes contributing to coatcolour in native Korean Hanwoo cattle. Animal Genet.

[14] Schmutz SM, Dreger DL (2012). Interaction of MC1R and PMEL alleles on solid coat colours in Highland cattle. Animal Genet..

[15] Seitz JJ, Schmutz SM, Thue TD, Buchanan FC (1999). A missense mutation in the bovine MGF gene is associated with the roan phenotype in Belgian blue and shorthorn cattle. Mamm Genome.

[16] Philipp U, Lupp B, Moemke S, Stein V, Tipold A, Eule JC (2011). A MITF mutation associated with a dominant white phenotype and bilateral deafness in German Fleckvieh cattle. PLoS One.

[17] Hulsman Hanna LL, Sanders JO, Riley DG, Abbey CA, Gill CA (2014). Identification of a major locus interacting with MC1R and modifying black coat colour in an F2 Nellore-Angus population. Genet Select Evol.

[18] Fontanesi L, Rustempasic A, Brka M, Russo V (2012). Analysis of polymorphisms in the agouti signalling protein (ASIP) and melanocortin 1 receptor (MC1R) genes and association with coat colours in two Pramenka sheep types. Small Ruminant Res.

[19] Fontanesi L, Beretti F, Riggio V, Gomez Gonzales E, Dall’Olio S, Davoli R (2008). Copy number variation and missense mutations of the Agouti signaling protein (ASIP) gene in goat breeds with different coat colours. Cytogenet Genome Res.

[20] Miao YW, Wu GS, Wang L, Li DL, Tang SK, Liang JP (2010). The role of MC1R gene in buffalo coat colour. Sci China Life Sci.

[21] Reiner G, Tramberend K, Nietfeld F, Volmer K, Wurmser C, Fries R, et al. A genome-wide scan study identifies a single nucleotide substitution in the tyrosinase gene associated with white coat colour in a red deer (*Cervus elaphus*) population. BMC Genet. 10.1186/s12863-020-0814-0. 10.1186/s12863-020-0814-0 PMC7011275 32041521

[22] Kuehn R, Anastassiadis C, Pirchner F. Transfer of bovine microsatellites to the cervine (*Cervus elaphus*). Animal Genet. 1996;27:199–201. 10.1111/j.1365-2052.1996.tb00952.x 8759122

[23] Slate J, Coltman DW, Goodman SJ, MacLean I, Pemberton JM, Williams JL. Bovine microsatellite loci are highly conserved in red deer (*Cervus elaphus*), sika deer (Cervus nippon) and Soay sheep (Ovis aries). Animal Genet. 1998;29:307–315. 10.1046/j.1365-2052.1998.00347.x 9745670

[24] Bana NA, Nyira A, Nagy J, Frank K, Nagy T, Steger V, et al. The red deer *Cervus elaphus* genome CerEla1.0: sequencing, annotating, genes, and chromosomes. Mol Genet Genomics 2018;293:665–684. 10.1007/s00438-017-1412-3 29294181

[25] Herraiz C, Garcia-Borron JC, Jiminez-Cervantes C, Olivares C (1863). MC1R signalling. Intracellular partners and pathophysiological implications. Biochim Biophys Acta.

[26] Saleha S, Khan TA, Zafar S (2016). MC1R gene variants involvement in human OCA phenotype. Open Life Sci.

[27] Robbins LS, Nadeau JH, Johnson KR, Kelly MA, Rosellirehfuss L, Baack E, Mountjoy KG, Cone RD (1993). Pigmentation phenotypes of variant extension locus alleles result from point mutations that alter MSH receptor function. Cell..

[28] Marklund L, Moller MJ, Sandberg K (1996). Andersson L (1996) a missense mutation in the gene for melanocyte-stimulating hormone receptor (MC1R) is associated with the chestnut coat color in horses. Mamm Genome.

[29] Vage DI, Lu D, Klungland H, Lien S, Adalsteinsson S, Cone RD (1997). A non-epistatic interaction of agouti and extension in the fox (Vulpes vulpes). Nature Gen.

[30] Newton JM, Wilkie AL, He L, Jordan SA, Metallinos DL, Holmes NG, Jackson IJ, Barsh GS (2000). Melanocortin 1 receptor variation in the domestic dog. Mamm Genome.

[31] Fontanesi L, Scotti E, Colombo M, Beretti F, Forestier L, Dall’Olio S, Deretz S, Russo V, Allain D, Oulmouden A (2010). A composite six bp in-frame deletion in the melanocortin 1 receptor (MC1R) gene is associated with the Japanese grindling coat color in rabbits (Oryctolagus cuniculus). BMC Genet.

[32] Guo XL, Li XL, Li Y, Gu ZL, Zheng CS, Wei ZH, Wang JS, Zhou RY, Li LH (2010). Zheng HQ (2010) genetic variation of chicken MC1R gene in different plumage color populations. Br Poult Sci.

[33] Chandramohan B, Renieri C, La Manna V, La Terza A. The Alpaca melanocortin 1 receptor: gene mutations, transcrpts, and relative levels of expression in ventral skin biopsies. Hindawi Publ Corp Sci World J. 2015;ID 265751. 10.1155/2015/265751 PMC4313674 25685836

[34] Fontanesi L, Beretti F, Riggio V, Dall’Olio S, Calascibetta D, Russo V, Portolano B (2010). Sequence characterization of the melanocortin 1 receptor (MC1R) gene in sheep with different coat colors and identification of the putative e allele at the ovine extension locus. Small Ruminant Res..

[35] Yang GL, Fu DL, Lang X, Yan YF, Luo YZ (2014). Genetic variation of 5 SNPs of MC1R gene in Chinese indigenous sheep breeds. Russ J Gen.

[36] Kırıkc K, Nocea A, Zidi A, Serradilla JM, Carrizosac J, Urrutiac B, Pilla F, D’Andread M, Capotee J, Bizelis I, Balteanug V, Figueiredo Cardosoa T, Eghbalsaiedi S, Ponsj A, Álvarezk LA, Pazzolal M, Vaccal GM, Obexer-Ruffm G, Amillsa M (2016). Analysing the diversity of the caprine melanocortin 1 receptor (MC1R) in goats with distinct geographic origins. Small Ruminant Res..

[37] Klungland H, Vage DI, Gomez-Raya L, Adalsteinsson S, Lien S (1995). The role of melanocyte stimulating hormone (MSH) receptor in bovine coat color determination. Mamm Genome.

[38] Zhang Y, Li Q, Ye S, Faruque MO, Yu Y, Sun D, Zhang S, Wang Y (2014). New variants in the melanocortin 1 receptor gene (MC1R) in Asian cattle. Animal Gen.

[39] Vage DI, Fuglei E, Snipstad K, Beheim J, Landsem VM (2005). Klungland H (2005) two cysteine substitutions in the MC1R generate the blue variant of the arctic fox (Alopex lagopus) and prevent expression of the white winter coat. Peptides..

[40] Vage DI, Nieminen M, Anderson DG, Roed KH (2014). Two missense mutations in melanocortin 1 receptor (MC1R) are strongly associated with dark ventral coat color in reindeer (Rangifer tarandus). Animal Gen..

[41] Suzuki H (2013). Evolutionary and phylogeographic views on Mc1r and Asip variation in mammals. Genes Genet Syst..

[42] Ritland K, Newton C, Marshall HD (2001). Inheritance and population structure of the white-phased Kermode black bear. Curr Biol.

[43] Kerje S, Lind J, Schütz K, Jensen P, Andersson L (2003). Melanocortin 1 receptor (MC1R) mutations are associated with plumage color in chicken. Anim Genet.

[44] Hosoda T, Sato JJ, Shimada T, Campbell KL, Suzuki H (2005). Independent nonframeshift deletions in the MC1R gene are not associated with melanistic coat coloration in three mustelid lineages. J Hered.

[45] Shimada T, Sato JJ, Aplin KP, Suzuki H (2009). Comparative analysis of evolutionary modes in Mc1r coat colour gene in wild mice and mustelides. Genes Genet Syst.

[46] Dürig N, Letko A, Lepori V, Hadji Rasouliha S, Loechel R, Kehl A, Hytönen MK, Lohi H, Mauri N, Dietrich J, Wiedmer M, Drögemüller M, Jagannathan V, Schmutz SM, Leeb T (2018). Two MC1R loss-of function alleles in cream-colored Australian cattle dogs and white huskies. Animal Gen.

[47] Almathen F, Elbir H, Bahbahani H, Mwacharo J, Hanotte O (2018). Polymorphisms in MC1R and ASIP genes are associated with coat color variation in the Arabian camel. J Heredity.

[48] Cooksey CJ, Garratt PJ, Land EJ, Pavel S, Ramsden CA, Riley PA (1997). Evidence oft he indirect formation of the catecholic intermediate substrate responsible for the autoactivation kinetics of tyrosinase. J Biol Chem.

[49] Perez-Oliva AB, Olivares C, Jimenez-Cervantes C, Garcia-Borron JC (2008). Mahogunin ring finger-1 (MGRN1) E3 ubiquitin ligase inhibits signalling from melanocortin receptor by competition with Galphas. J Biol Chem.

[50] Jimenez-Cervantes C, Germer S, Gonzales P, Sanchez J, Sanchez CO, Garcia-Borron JC (2001). Thr40 and Met122 are new partial loss-of-function natural mutations of the human melanocortin 1 receptor. FEBS Lett.

[51] Schioeth HB, Phillips SR, Rudzish R, Birch-Machin MA, Wikberg JES, Rees JL (1999). Loss of function mutation of the human melanocortin 1 receptor are common and are associated with red hair. Biochim Biophys Res Commun.

[52] Johnson CP, Gaetani M, Ortiz V, Bhasin N, Harper S, Gallagher PG, Speicher DW, Discher DE (2019). Pathogenic proline mutation in the linker between spectrin repeats: disease caused by spectrin unfolding. Blood..

[53] Chou PY, Fasman GD (1974). Prediction of protein conformation. Biochemistry..

[54] Khan S, Vihinen M (2007). Spectrum of disease-causing mutations in protein secondary structures. BMC Structural Biol.

[55] Zimin AV, Delcher AL, Florea L, Kelley DR, Schatz MC, Puiu D, et al. A whole-genome assembly of the domestic cow, *Bos taurus*Genome Biol 2009;10:R42. 10.1186/gb-2009-10-4-r42 PMC2688933 19393038

[56] Li H, Handsaker B, Wysoker A, Fennell T, Ruan J, Homer N (2009). The sequence alignment/map format and SAMtools. Bioinformatics..

[57] McLaren W, Gil L, Hunt SE, Riat HS, Ritchie GRS, Thormann A (2016). The Ensembl variant effect predictor. Genome Biol.

