# Supplementary material for: A genome-wide scan study identifies a single nucleotide substitution in MC1R gene associated with white coat colour in fallow deer (Dama dama)
Source: BMC Genet. 2020 Nov 19;21:126. doi: 10.1186/s12863-020-00950-3 (PMC7678172; doi:10.1186/s12863-020-00950-3)
Supplement: Supplementary file 1 — Additional file 1: Supplementary Table S1. Genotype-phenotype associations in white and brown fallow deer for all tested single nucleotide variants. [file 12863_2020_950_MOESM1_ESM.docx]

Supplemtary File

**A genome-wide scan study identifies a single nucleotide substitution in *MC1R* gene associated with white coat colour in fallow deer (*Dama dama*)**

Gerald Reiner, Tim Weber, Florian Nietfeld, Dominik Fischer, Christine Wurmser, Ruedi Fries and Hermann Willems

Supplementary Table 1: Genotype-phenotype associations in white and brown fallow deer for all tested single nucleotide variants.

| Chr | Chr_pos | Gene | Hind (white) | Calf (brown) | White1 | Brown1 | White2 | White3 | White4 | Brown2 | Brown3 | Brown4 |
| --- | --- | --- | --- | --- | --- | --- | --- | --- | --- | --- | --- | --- |
| 13 | 51640322 | *ATRN* | **GG** | **GA** | AA | GG | *na* | *na** | *na* | *na* | *na* | *na* |
| 23 | 41483931 | *DTNBP1* | **GG** | **GC** | CC | **CC** | *na* | *na* | *na* | *na* | *na* | *na* |
| 26 | 22870530 | *ELOVL3* | **AA** | **AG** | GG | **GG** | *na* | *na* | *na* | *na* | *na* | *na* |
| 12 | 23298094 | *FREM2* | **GG** | **GA** | AA | GG | *na* | *na* | *na* | *na* | *na* | *na* |
| 12 | 23336198 | *FREM2* | **CC** | **CA** | **CC** | **CA** | ***CC*** | CA | **CC** | **AA** | CC | ***AA*** |
| 17 | 71465091 | *GGT1* | **AA** | **AG** | GG | **GG** | *na* | *na* | *na* | *na* | *na* | *na* |
| 28 | 4036781 | *GNPAT* | **GG** | **GA** | **GG** | GG | *na* | *na* | *na* | *na* | *na* | *na* |
| X | 37080066 | *L1CAM* | **GG** | **GA** | AA | **AA** | *na* | *na* | *na* | *na* | *na* | *na* |
| 28 | 8475854 | *LYST* | **CC** | **AC** | **CC** | **AC** | ***CC*** | **CC** | AC | **AC** | **AC** | ***AC*** |
| 18 | 14705518 | *MC1R* | **CC** | **TC** | **CC** | **TC** | ***CC*** | **CC** | **CC** | **TT** | **TT** | ***TT*** |
| 11 | 103981017 | *NOTCH1* | **AA** | **AC** | CC | **CC** | *na* | *na* | *na* | *na* | *na* | *na* |
| 3 | 13984639 | *NTRK1* | **AA** | **AG** | GG | **GG** | *na* | *na* | *na* | *na* | *na* | *na* |
| 5 | 57353185 | *PMEL* | **TT** | **TC** | **TT** | TT | *na* | *na* | *na* | *na* | *na* | *na* |
| 14 | 429568 | *RECQL4* | **AA** | **AG** | **AA** | AA | *na* | *na* | *na* | *na* | *na* | *na* |
| 14 | 429631 | *RECQL4* | **TT** | **TC** | CC | **CC** | *na* | *na* | *na* | *na* | *na* | *na* |
| 4 | 40075565 | *SEMA3C* | **GG** | **GA** | AA | **AA** | *na* | *na* | *na* | *na* | *na* | *na* |
| 3 | 14622613 | *SEMA4A* | **GG** | **GC** | **GG** | **GC** | ***GG*** | GC | **GG** | GG | GG | *GG* |
| X | 133162026 | *SHROOM2* | **CC** | **CA** | AA | **AA** | *na* | *na* | *na* | *na* | *na* | *na* |
| X | 133162029 | *SHROOM2* | **CC** | **CT** | TT | **TT** | na | na | na | na | na | na |

Genotypes in bold indicate corresponding genotype-phenotype associations. Hind and calf: the sequenced individuals; White1 through White4 and Brown1 through Brown4 indicate different white and brown fallow deer used for genotyping; White1 and Brown1 were used on the first run, White and Brown 2-4 on the second run; na: not analysed.
